# Variation in the human cannabinoid receptor *CNR1 *gene modulates gaze duration for happy faces

**DOI:** 10.1186/2040-2392-2-10

**Published:** 2011-06-29

**Authors:** Bhismadev Chakrabarti, Simon Baron-Cohen

**Affiliations:** 1 Centre for Integrative Neuroscience and Neurodynamics, School of Psychology and Clinical Language Sciences, University of Reading, Whiteknights, Reading RG6 6AL, UK; 2 Autism Research Centre, Department of Psychiatry, University of Cambridge, Douglas House, 18B Trumpington Road, Cambridge CB2 8AH, UK

## Abstract

**Background:**

From an early age, humans look longer at preferred stimuli and also typically look longer at facial expressions of emotion, particularly happy faces. Atypical gaze patterns towards social stimuli are common in autism spectrum conditions (ASC). However, it is unknown whether gaze fixation patterns have any genetic basis. In this study, we tested whether variations in the cannabinoid receptor 1 (*CNR1*) gene are associated with gaze duration towards happy faces. This gene was selected because *CNR1 *is a key component of the endocannabinoid system, which is involved in processing reward, and in our previous functional magnetic resonance imaging (fMRI) study, we found that variations in *CNR1 *modulate the striatal response to happy (but not disgust) faces. The striatum is involved in guiding gaze to rewarding aspects of a visual scene. We aimed to validate and extend this result in another sample using a different technique (gaze tracking).

**Methods:**

A total of 30 volunteers (13 males and 17 females) from the general population observed dynamic emotional expressions on a screen while their eye movements were recorded. They were genotyped for the identical four single-nucleotide polymorphisms (SNPs) in the *CNR1 *gene tested in our earlier fMRI study.

**Results:**

Two SNPs (rs806377 and rs806380) were associated with differential gaze duration for happy (but not disgust) faces. Importantly, the allelic groups associated with a greater striatal response to happy faces in the fMRI study were associated with longer gaze duration at happy faces.

**Conclusions:**

These results suggest that *CNR1 *variations modulate the striatal function that underlies the perception of signals of social reward, such as happy faces. This suggests that *CNR1 *is a key element in the molecular architecture of perception of certain basic emotions. This may have implications for understanding neurodevelopmental conditions marked by atypical eye contact and facial emotion processing, such as ASC.

## Background

Vision is the primary sensory modality in primates, reflected by the visual cortex being the largest of all the sensory cortices. Our eyes perform quick orienting movements ('saccades') towards interesting features of stimuli in the external world [1]. In general, we tend to look longer at more rewarding stimuli [2]. This rationale lies behind the 'preferential looking' technique in infancy research, where gaze duration and direction are assumed to reflect visual preference [2-6]. Gaze not only informs us about normative variation in the visual processing of stimuli but also is relevant to the understanding of complex neurodevelopmental conditions such as autism spectrum conditions (ASC), which are characterised by atypical gaze fixation patterns [7,8]. This has led to the suggestion that gaze fixation patterns could constitute potential endophenotypes for such conditions. Gaze patterns show high test-retest reliability as well as a moderate to high heritability when tested in twins [9-11], suggesting a significant genetic contribution. This raises the possibility that variation in candidate genes underlie normative variation in gaze patterns.

The measure of particular interest to us is the duration of gaze fixation, given the evidence that people with ASC show reduced gaze fixation towards social stimuli [8,12-15]. Research in primates suggests that the striatal region plays a major role in directing gaze [16]. The striatum is thought to encode a 'value map' of the visual stimuli. Both ventral striatal neurons as well as a subpopulation of caudate neurons encode reward magnitude of the stimuli [17,18]. This 'value map', in addition to further frontal cortical inputs, is then passed to the lateral intraparietal area (LIP), where a fine-tuned map of 'relative expected subjective value' is created. The LIP projects to the frontal eye fields, which send excitatory projections to the caudate nucleus. A subset of neurons from the caudate nucleus inhibit the substantia nigra and consequently disinhibit the superior colliculus, which in turn controls the gaze control nuclei in the brainstem, leading to a gaze shift [19].

One of the key molecular systems involved in the functioning of the striatal circuit is the endocannabinoid system. It is a neuropeptidergic circuit involved in reward processing and works in tandem with the mesolimbic dopaminergic system [20]. Expressed selectively in the brain, the cannabinoid receptor 1 (CNR1) is the best-studied molecule of this system. Immunolocalisation studies in rats and humans have indicated high *CNR1 *expression levels in the striatum, a region known for its central role in reward processing [20-24]. CNR1 is believed to modulate striatal dopamine release through a trans-synaptic mechanism involving both GABAergic and glutamatergic synapses and is expressed strongly in the caudate, putamen, globus pallidus internal and substantia nigra, as well as in the shell of the nucleus accumbens [25]. Phasic release of striatal dopamine is the primary mechanism encoding for reward [26].

Recent studies have suggested abnormalities in ASC in striatal volume [27,28], connectivity [29] and activity in response to social stimuli [30]. In addition, a gene expression study of postmortem brain tissue of people with ASC found reduced expression of *CNR1 *[31]. In view of the atypical gaze behaviour of people with ASC, together with the observed striatal atypicalities, it is reasonable to examine the phenotype of gaze patterns as a function of variation in genes expressed in the striatum.

As gaze fixation is linked to striatal activity [16,17,19], we might expect that molecular variation in the genes involved in striatal function would be associated with differences in gaze towards socially rewarding stimuli. Using functional magnetic resonance imaging (fMRI), we previously found genetic variation in *CNR1 *modulated activity in the striatal region while watching happy (but not disgust) faces [32]. This result has been independently replicated in larger samples [33]. In the current study, we aimed to conduct an identical experiment using gaze-tracking in a new sample of volunteers. Specifically, we tested whether *CNR1 *genetic variation influences gaze duration towards happy faces. To ensure that this was closely matched to the original fMRI experiment, we also analysed gaze fixation duration for disgust expressions as a function of *CNR1 *genetic variation. Disgust faces are potential signals of 'nonreward', in contrast to rewarding happy faces, and hence provide a high-level control condition (that is, matched for face-specific qualities, such as configural features, as well as more general visual qualities of the stimuli, such as colour, shape and luminosity) for our experiment. We predicted that variation (single-nucleotide polymorphisms (SNPs)) in the *CNR1 *gene would be significantly associated with individual variability in gaze duration towards happy but not disgust faces.

## Methods

### Participants

A total of 30 student volunteers (13 males and 17 females; 29 right-handed and 1 left-handed; mean age ± SD, 24.1 ± 3.41 years old) were recruited by advertisement from the local universities. Participants were included only if all four grandparents were of Caucasian European ancestry to avoid genetic heterogeneity between different populations. Participants were also excluded if they reported any history of psychiatric diagnoses or regular drug abuse. They were equated for educational background in that all had completed high school and were studying towards a college degree. All had normal (or corrected to normal) vision. The study was approved by the Psychology Research Ethics Committee of the University of Cambridge.

**Figure 1:**
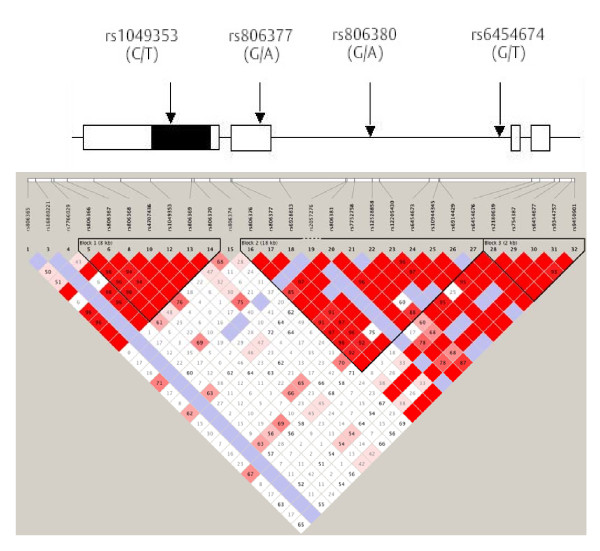
**Schematic structure of the cannabinoid receptor *CNR1 *gene with all four genotyped single-nucleotide polymorphisms indicated**. Top: White boxes indicate untranslated regions, black boxes indicate translated regions and intervening straight line indicates an intronic region. Bottom: The linkage disequilibrium structure of the gene in the Caucasian (CEU) population is shown (using the publicly available HapMap version 3, release R2, database available at http://hapmap.ncbi.nlm.nih.gov/.

Buccal swabs were collected from all participants, and DNA was extracted. The four SNPs of choice were identical to those selected in our earlier fMRI study (rs1049353, rs806377, rs806380 and rs6454674), chosen to ensure a minor allele frequency > 0.2 in a Caucasian population and to cover as much of the gene as possible (see Figure 1) [32]. The DNA was genotyped by Geneservice, Inc. (Cambridge, UK) using standard TaqMan™ assays (Applied Biosystems, Inc., California, USA). Genotyping for these SNPs failed for two of these participants, resulting in a sample size of 28 participants for the final analysis.

### Procedure

The stimuli were taken from the *Mindreading*™ set developed by Baron-Cohen *et al*. [34], since dynamic facial expressions of emotion are assumed to be more ecologically valid than static photographs. The *Mindreading *set consists of video, audio and textual examples for 412 different emotions arranged into 24 emotion groups and organised according to six different developmental levels (based on emotions recognised in childhood through adulthood). These stimuli have been validated in typical populations and in people with ASC [35-38]. These stimuli were chosen over other existing available stimuli because *Mindreading *stimuli comprise dynamic emotional expressions whilst alternatives (such as the Ekman and Friesen set [39], the Karolinska Directed Emotional Faces set [40] and the NimStim set [41]) comprise static expressions. The *Mindreading *stimuli have excellent interrater reliability and external validity [36,38] (stimuli are available at http://www.jkp.com/mindreading/).

Participants were seated comfortably at a fixed distance of 60 cm from the screen and were instructed to keep movement to a minimum. Participants watched 80 videoclips (three seconds each and sixteen clips for each of the five emotions) presented in a pseudorandom order using GazeTracker™ software (DynaVox Inc., Virginia, USA) with an interstimulus interval of six seconds. Participants were shown a fixation cross during the interstimulus interval. All stimuli were centred on a 19-inch monitor and occupied 70% of the screen area. To ensure that participants were attending to the stimulus, they were asked to say aloud what emotion they thought was being displayed (choosing one of five emotion words: 'happy', 'sad', 'angry', 'disgust' or 'fear'). Their responses were recorded by the experimenter.

**Figure 2:**
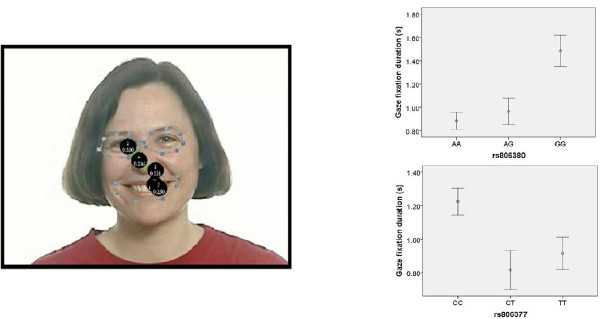
**Example gaze trail from a single participant and group mean gaze durations for happy faces classified by genotype**. **(a) **Example gaze trail from a single participant on a still frame from a video stimulus showing a happy expression. The black circles represent fixation points and the amount of time (in seconds) spent in each. The dotted lines demarcate each look zone (eyes region and mouth region). **(b) **Gaze duration for happy faces grouped by genotype for rs806380 (top) and rs806377 (bottom), respectively. Unfilled circles indicate mean gaze duration, and error bars represent ± 1 SEM. From the *Mindreading*™ set developed by Baron-Cohen *et al*. [34]

The Eye Response Interface Computer Aid camera (ERICA; http://www.eyeresponse.com/) was used to measure fixation time at each point at 60 Hz. ERICA uses reflected low-frequency infrared rays (λ = 880 nm) to map macrosaccades and fixation times at each point. The data were preprocessed using GazeTracker™ software. To ensure that the measured gaze duration was specific to the socially informative regions of the emotion expressions [35], 'look zones' were manually drawn around the eyes (the eyebrows and lower eyelids) and mouth region (the region from the bottom of the nose to the bottom of the lower lip) of all stimuli (see Figure 2). All look zones were 'dynamic'; that is, they tracked the eyes and the mouth regions while allowing for head movement of the actors.

The sum of duration of all fixations was recorded for each look zone. A fixation was defined as a continuous gaze for 100 ms within a 40-pixel diameter (corresponding to a 1.3° visual angle), which was in line with parameters used in similar studies [15]. Gaze duration for each expression was calculated by summing the mean fixation time for eyes and mouth regions. Different regions of the face (that is, eyes and mouth) are relevant for processing different basic emotions [12,42-44]. Thus it is not ideal to compare the fixation time to the eyes region for happy and disgust faces, since disgust faces are associated with greater gaze duration to the mouth region. Hence, total fixation time across eyes and mouth regions was used as the dependent variable.

## Results

Both happy and disgust expressions were recognised with > 80% accuracy. Genetic association was measured using the UNPHASED programme (http://www.mrc-bsu.cam.ac.uk/personal/frank/software/unphased), which computes the retrospective likelihood, that is, the probability of observing different genotypes given an observed distribution of a quantitative trait [45]. The two dependent variables (gaze duration for all happy and all disgust faces) and the genotypes for all four SNPs were included in a single analysis. This analysis revealed a significant association of the gaze duration for happy faces with rs806377 (χ^2 ^= 8.88, *df *= 2, *P *= 0.011) and rs806380 (χ^2 ^= 8.46, *df *= 2, *P *= 0.014). No significant associations (at *P *≤ 0.05) were noted for gaze duration for disgust faces (nominal p_rs806377 _= 0.104 and nominal p_rs806380 _= 0.086). To test whether the observed lack of significant association with disgust faces was due to one video clip that was misclassified by a majority of the participants, the data were reanalysed after removing all fixation data associated with this one video clip. This revealed an identical pattern of results, with a nominal p_rs806377 _= 0.111 and a nominal p_rs806380 _= 0.105.

However, when multiple SNPs are in linkage disequilibrium (LD), hypothesis tests in single-locus analyses are not independent. To take this into account, Li and Ji [46] proposed a method for estimating the true number of independent tests (*M*_eff_), which takes into consideration the LD between SNPs. This method was implemented using the SNPSpD software programme [47], which revealed that *M*_eff _was 3 in the current sample. The Bonferroni correction for three independent tests gave a corrected *P *= 0.033 for rs806377 and a corrected *P *= of 0.042 for rs806380 for association with gaze duration for happy faces.

To further analyse genotypic differences for each SNP that were significantly associated with gaze duration for happy faces, *post hoc t*-tests were conducted. In rs806377, the CC genotype was associated with longer gaze duration than the CT genotype (*t *= 2.92, *P *< 0.025 with the Bonferroni correction). In rs806380, the GG genotype was associated with longer gaze duration than the AA genotype (*t *= 2.78, *P *< 0.05 with the Bonferroni correction) (see Figure 2).

The main effects of all possible haplotypes were tested with various possible window sizes (two, three and four marker combinations) using UNPHASED software. None of these haplotypic association tests were significant at *P *< 0.05. While the small sample size did not allow for a robust test of sex differences in this genetic association, we report the nominal *P *values for these tests for the sake of completeness. rs806377 was significantly associated with the gaze duration for happy faces in both females (*P *= 0.021) and males (*P *= 0.004). Additionally, in males, rs806380 (*P *= 0.019) and rs1049353 (*P *= 0.004) were found to be associated with gaze duration for happy faces.

## Discussion

In this experiment, we predicted that *CNR1 *genetic variations would be associated with differences in gaze fixation duration towards happy faces. This prediction was confirmed: two SNPs in this gene (rs806377 and rs806380) were associated with differences in gaze duration for happy (but not disgust) faces. This finding fits well with the established role of the *CNR1 *gene in reward processes [20] and is consistent with the results of fMRI studies [32,33] in showing that this gene is a component of the molecular architecture of social reward processing. Social reward processing has been suggested to be impaired in people with ASC [48-50], particularly as reflected in atypical gaze patterns towards social stimuli. Hence the current results could be relevant to understanding the genetic underpinning of the social behavioural symptoms in people with ASC.

A comparison of these results with those from our earlier fMRI study reveals that for the SNP rs806377, the allelic group (CC) associated with the highest striatal response is also associated with the longest gaze duration for happy faces. For rs806380, the allelic group associated with the highest striatal response (GG) is also associated with the longest gaze duration for happy faces. rs806377 is located in an untranslated region (UTR) of the gene (Figure 1), and rs806380 was found to be in significant LD with a 5'-UTR SNP (rs78074274) using CandiSNPer [51]. The observed effects can thus be mediated by either or both of these UTR SNPs by potentially altering gene transcription and/or mRNA stability. Since the fMRI and gaze duration data come from largely independent samples (only three of thirty participants were common to both studies), it is likely that the observed genetic differences reflect real effects.

We interpret the genetically linked biasing of visual perception in terms of individual differences in the reward circuitry. The two processes of increased visual preference (indicated by longer gaze duration) and increased striatal response for happy faces are linked in a positive feedback loop [5]. We tend to look longer at preferred stimuli, which in turn increases our preference/'reward value' for these stimuli. Consequently, we interpret the observed effect in biasing visual perception of social stimuli in terms of differences in the individual reward circuitry. Whether such intrinsic differences in reward circuitry change the formation and nature of 'saliency/value maps' formed during gaze fixation is a question for future research [19,52].

A second broader question for future research is whether the observed *CNR1 *genotypic differences in fixation duration for happy faces is specific to social rewards or whether this holds true for all classes of rewards. Variation in *CNR1 *has been linked to polysubstance abuse and associated with increased activity in reward-processing areas of the brain in response to drug cues for both marijuana and alcohol addicts [53,54]. Hence it is possible that the observed genotypic differences in the general population may extend to other classes of rewards. Crucially, however, a reduced experience of rewards in response to social stimuli such as happy faces (as has been suggested by Dawson *et al*. [48] to apply to ASC) has more far-reaching consequences, since if an infant is looking less at happy faces and is finding them less rewarding, this will make social interactions less reinforcing, which in turn can exacerbate the social difficulties observed in people with ASC.

It is possible that a number of genes, each of small to medium effect size, determine the striatal response to social stimuli such as happy faces. Other potential candidate genes might include those involved in the oxytocin-vasopressin system (*OXTR, AVPR1A *and *AVPR1B*) as well as those coding for key proteins involved in neutrotransmission (for example, *MAOA *and *GABRB3*) [55]. We speculate that these genes have an additive effect and might potentially underlie complex traits related to social functioning [56]. In a larger population-based genetic association study of empathy, we found a nominally significant association of *CNR1 *genetic variation with the Empathy Quotient [55,57]. Additionally, reduced expression of *CNR1 *was found in postmortem brain tissue of individuals with ASC [31]. Together, these findings further support the implication that variation in the *CNR1 *gene modulates the response to social stimuli such as happy faces.

However, the current findings should be interpreted with caution, since, in the absence of any expression data, any functional role for the SNPs can only be speculative; that is, the observed SNP effects may be caused by being in LD with other functional polymorphisms and/or through mechanisms that affect mRNA stability or splicing as mentioned earlier. However, the observation of genetic differences in two separate (albeit small) samples using an identical paradigm with two different techniques points towards a putative role played by *CNR1 *in the response to happy faces.

## Conclusions

In this study, we tested whether common variants in the *CNR1 *gene modulate gaze duration towards happy faces. We found that two SNPs in this gene were significantly associated with gaze duration for happy (but not disgust) faces. This result is consistent with that of our previous fMRI study [32]. Specifically, the allelic groups that were found to be associated with the strongest striatal response in our fMRI study were associated with the longest gaze duration for happy faces in the current sample. This finding suggests a role for *CNR1 *in social reward processing and could have significance for clinical conditions such ASC, which are marked by a deficit in social reward processing as well as atypical responses to facial expressions of emotion [35,36,49].

## Abbreviations

*CNR1*: cannabinoid receptor 1; mRNA: messenger RNA; SNP: single-nucleotide polymorphism.

## Competing interests

The authors declare that they have no competing interests.

## Authors' contributions

BC designed and ran the experiment, analysed the data and wrote the paper. SBC provided intellectual input at all stages and cowrote the paper. Both authors read and approved the final manuscript.

## References

[B1] Moschovakis A Highstein SM The anatomy and physiology of primate neurons that control rapid eye movements Annu Rev Neurosci 1994 17 465 488 10.1146/annurev.ne.17.030194.002341 8210184

[B2] Calvo M Avero P Time course of attentional bias to emotional scenes in anxiety: gaze direction and duration Cogn Emot 2005 19 433 451 10.1080/02699930441000157 22686651

[B3] Butterworth G Jarrett N What minds have in common is space: spatial mechanisms serving joint visual attention in infancy Br J Dev Psychol 1991 9 55 72

[B4] Calvo M Lang P Gaze patterns when looking at emotional pictures: motivationally biased attention Motiv Emot 2004 28 221 243

[B5] Shimojo S Simion C Shimojo E Scheier C Gaze bias both reflects and influences preference Nat Neurosci 2003 6 1317 1322 10.1038/nn1150 14608360

[B6] Connellan J Baron-Cohen S Wheelwright S Batki A Ahluwalia J Sex differences in human neonatal social perception Infant Behav Dev 2001 23 113 118

[B7] Fletcher-Watson S Leekam SR Benson V Frank MC Findlay JM Eye-movements reveal attention to social information in autism spectrum disorder Neuropsychologia 2009 47 248 257 10.1016/j.neuropsychologia.2008.07.016 18706434

[B8] Klin A Jones W Schulz R Volkmar F Cohen DJ Visual fixation patterns during viewing of naturalistic social situations as predictors of social competence in individuals with autism Arch Gen Psychiatry 2002 9 809 816 10.1001/archpsyc.59.9.809 12215080

[B9] Katsanis J Taylor J Iacono WG Hammer MA Heritability of different measures of smooth pursuit eye tracking dysfunction: a study of normal twins Psychophysiology 2000 37 724 730 10.1111/1469-8986.3760724 11117452

[B10] Iacono WG Eye tracking in normal twins Behav Genet 1982 12 517 526 10.1007/BF01073782 6892006

[B11] Greenwood TA Braff DL Light GA Cadenhead KS Calkins ME Dobie DJ Freedman R Green MF Gur RE Gur RC Mintz J Nuechterlein KH Olincy A Radant AD Seidman LJ Siever LJ Silverman JM Stone WS Swerdlow NR Tsuang DW Tsuang MT Turetsky BI Schork NJ Initial heritability analyses of endophenotypic measures for schizophrenia: the Consortium on the Genetics of Schizophrenia Arch Gen Psychiatry 2007 64 1242 1250 10.1001/archpsyc.64.11.1242 17984393 PMC10588564

[B12] Baron-Cohen S Wheelwright S Jolliffe T Is there a "language of the eyes"? Evidence from normal adults and adults with autism or Asperger syndrome Vis Cogn 1997 4 311 331 10.1080/713756761

[B13] Joliffe T Mortimore C Robertson M Another advanced test of theory of mind: evidence from very high functioning adults with autism or Asperger syndrome J Child Psychol Psychiatry 1997 38 813 822 10.1111/j.1469-7610.1997.tb01599.x 9363580

[B14] Swettenham J Baron-Cohen S Charman T Cox A Baird G Drew A Rees L Wheelwright S The frequency and distribution of spontaneous attention shifts between social and non-social stimuli in autistic, typically developing, and non-autistic developmentally delayed infants J Child Psychol Psychiatry 1998 9 747 753 9690937

[B15] Senju A Southgate V White S Frith U Mindblind eyes: an absence of spontaneous theory of mind in Asperger syndrome Science 2009 325 883 885 10.1126/science.1176170 19608858

[B16] Hikosaka O Basal ganglia mechanisms of reward-oriented eye movement Ann N Y Acad Sci 2007 1104 229 249 10.1196/annals.1390.012 17360800

[B17] Lau B Glimcher PW Action and outcome encoding in the primate caudate nucleus J Neurosci 2007 27 14502 14514 10.1523/JNEUROSCI.3060-07.2007 18160658 PMC6673449

[B18] Cai X Kim S Lee D Heterogeneous coding of temporally discounted values in the dorsal and ventral striatum during intertemporal choice Neuron 2011 69 170 182 10.1016/j.neuron.2010.11.041 21220107 PMC3034314

[B19] Trommershäuser J Glimcher PW Gegenfurtner KR Visual processing, learning and feedback in the primate eye movement system Trends Neurosci 2009 32 583 590 10.1016/j.tins.2009.07.004 19729211

[B20] Gardner EL Vorel SR Cannabinoid transmission and reward-related events Neurobiol Dis 1998 5 502 533 10.1006/nbdi.1998.0219 9974181

[B21] Freund TF Katona I Piomelli D Role of endogenous cannabinoids in synaptic signaling Physiol Rev 2003 83 1017 1066 12843414 10.1152/physrev.00004.2003

[B22] Fusco FR Martorana A Giampà C De March Z Farini D D'Angelo V Sancesario G Bernardi G Immunolocalization of CB1 receptor in rat striatal neurons: a confocal microscopy study Synapse 2004 53 159 167 10.1002/syn.20047 15236348

[B23] Hurley MJ Mash DC Jenner P Expression of cannabinoid CB1 receptor mRNA in basal ganglia of normal and parkinsonian human brain J Neural Transm 2003 110 1279 1288 10.1007/s00702-003-0033-7 14628192

[B24] Haber SN Knutson B The reward circuit: linking primate anatomy and human imaging Neuropsychopharmacology 2010 35 4 26 10.1038/npp.2009.129 19812543 PMC3055449

[B25] van der Stelt M Di Marzo V The endocannabinoid system in the basal ganglia and in the mesolimbic reward system: implications for neurological and psychiatric disorders Eur J Pharmacol 2003 480 133 150 10.1016/j.ejphar.2003.08.101 14623357

[B26] Schultz W Getting formal with dopamine and reward Neuron 2002 36 241 263 10.1016/S0896-6273(02)00967-4 12383780

[B27] Langen M Schnack HG Nederveen H Bos D Lahuis BE de Jonge MV van Engeland H Durston S Changes in the developmental trajectories of striatum in autism Biol Psychiatry 2009 66 327 333 10.1016/j.biopsych.2009.03.017 19423078

[B28] Estes A Shaw DW Sparks BF Friedman S Giedd JN Dawson G Bryan M Dager SR Basal ganglia morphometry and repetitive behavior in young children with autism spectrum disorder Autism Res in press 10.1002/aur.193 PMC3110551 21480545

[B29] Di Martino A Kelly C Grzadzinski R Zuo XN Mennes M Mairena MA Lord C Castellanos FX Milham MP Aberrant striatal functional connectivity in children with autism Biol Psychiatry 2011 69 847 856 10.1016/j.biopsych.2010.10.029 21195388 PMC3091619

[B30] Weng SJ Carrasco M Swartz JR Wiggins JL Kurapati N Liberzon I Risi S Lord C Monk CS Neural activation to emotional faces in adolescents with autism spectrum disorders J Child Psychol Psychiatry 2011 52 296 305 10.1111/j.1469-7610.2010.02317.x 21039484 PMC3035431

[B31] Purcell A Jeon O Zimmerman A Blue M Pevsner J Postmortem brain abnormalities of the glutamate neurotransmitter system in autism Neurology 2001 57 1618 1628 11706102 10.1212/wnl.57.9.1618

[B32] Chakrabarti B Kent L Suckling J Bullmore E Baron-Cohen S Variations in the human cannabinoid receptor (*CNR1*) gene modulate striatal responses to happy faces Eur J Neurosci 2006 23 1944 1948 10.1111/j.1460-9568.2006.04697.x 16623851

[B33] Domschke K Dannlowski U Ohrmann P Lawford B Bauer J Kugel H Heindel W Young R Morris P Arolt V Deckert J Suslow T Baune BT Cannabinoid receptor 1 (*CNR1*) gene: impact on antidepressant treatment response and emotion processing in major depression Eur Neuropsychopharmacol 2008 18 751 759 10.1016/j.euroneuro.2008.05.003 18579347

[B34] Baron-Cohen S Golan O Wheelwright S Hill JJ Mindreading: The Interactive Guide to Emotions 2004 London: Jessica Kingsley Ltd

[B35] El Kaliouby R Robinson P Keates S Temporal context and the recognition of emotion from facial expression Proceedings of HCI International Conference 2003 Springer-Verlag

[B36] Golan O Baron-Cohen S Systemizing empathy: teaching adults with Asperger's syndrome/high functioning autism to recognize emotions using interactive multimedia Dev Psychopathol 2006 18 591 617 16600069 10.1017/S0954579406060305

[B37] Chakrabarti B Bullmore E Baron-Cohen S Empathizing with basic emotions: common and discrete neural substrates Soc Neurosci 2006 1 364 384 10.1080/17470910601041317 18633800

[B38] Golan O Baron-Cohen S Hill J The Cambridge Mindreading (CAM) Face-Voice Battery: testing complex emotion recognition in adults with and without Asperger syndrome J Autism Dev Disord 2006 36 169 183 10.1007/s10803-005-0057-y 16477515

[B39] Ekman P Friesen W Pictures of Facial Affect 1976 Palo Alto: Consulting Psychologists Press

[B40] Lundqvist D Flykt A Öhman A The Karolinska Directed Emotional Faces-KDEF, CD ROM from Department of Clinical Neuroscience, Psychology section, Karolinska Institutet 1998 Stockholm: Karolinska Institutet

[B41] Tottenham N Tanaka JW Leon AC McCarry T Nurse M Hare TA Marcus DJ Westerlund A Casey BJ Nelson C The NimStim set of facial expressions: judgments from untrained research participants Psychiatry Res 2009 168 242 249 10.1016/j.psychres.2008.05.006 19564050 PMC3474329

[B42] Chakrabarti B Individual differences in human emotion perception: neuroimaging, genetic and behavioural investigations PhD thesis 2007 Department of Psychiatry, School of Clinical Medicine, University of Cambridge

[B43] Gamer M Büchel C Amygdala activation predicts gaze toward fearful eyes J Neurosci 2009 29 9123 9126 10.1523/JNEUROSCI.1883-09.2009 19605649 PMC6665435

[B44] Kliemann D Dziobek I Hatri A Steimke R Heekeren HR Atypical reflexive gaze patterns on emotional faces in autism spectrum disorders J Neurosci 2010 30 12281 12287 10.1523/JNEUROSCI.0688-10.2010 20844124 PMC6633461

[B45] Dudbridge F Likelihood-based association analysis for nuclear families and unrelated subjects with missing genotype data Hum Hered 2008 66 87 98 10.1159/000119108 18382088 PMC2386559

[B46] Li J Ji L Adjusting multiple testing in multilocus analyses using the eigenvalues of a correlation matrix Heredity 2005 95 221 227 10.1038/sj.hdy.6800717 16077740

[B47] Nyholt DR A simple correction for multiple testing for SNPs in linkage disequilibrium with each other Am J Hum Genet 2004 74 765 769 10.1086/383251 14997420 PMC1181954

[B48] Dawson G Carver L Meltzoff AN Panagiotides H McPartland J Webb SJ Neural correlates of face and object recognition in young children with autism spectrum disorder, developmental delay, and typical development Child Dev 2002 73 700 717 10.1111/1467-8624.00433 12038546 PMC3651041

[B49] Scott-Van Zeeland AA Dapretto M Ghahremani DG Poldrack RA Bookheimer SY Reward processing in autism Autism Res 2010 3 53 67 20437601 10.1002/aur.122 PMC3076289

[B50] Kohls G Peltzer J Herpertz-Dahlmann B Konrad K Differential effects of social and non social reward on response inhibition in children and adolescents Dev Sci 2009 12 614 625 10.1111/j.1467-7687.2009.00816.x 19635087

[B51] Schmitt AO Aßmus J Bortfeldt RH Brockmann GA CandiSNPer: a web tool for the identification of candidate SNPs for causal variants Bioinformatics 2010 26 969 970 10.1093/bioinformatics/btq068 20172942

[B52] Henderson J Human gaze control during real-world scene perception Trends Cogn Sci 2003 7 498 504 10.1016/j.tics.2003.09.006 14585447

[B53] Filbey FM Schacht JP Myers US Chavez RS Hutchison KE Individual and additive effects of the *CNR1 *and *FAAH *genes on brain response to marijuana cues Neuropsychopharmacology 2009 35 967 975 20010552 10.1038/npp.2009.200 PMC2820137

[B54] Hutchison KE Haughey H Niculescu M Schacht J Kaiser A Stitzel J Horton WJ Filbey F The incentive salience of alcohol: translating the effects of genetic variant in *CNR1* Arch Gen Psychiatry 2008 65 841 850 10.1001/archpsyc.65.7.841 18606956 PMC2856651

[B55] Chakrabarti B Dudbridge F Kent L Wheelwright S Hill-Cawthorne G Allison C Banerjee-Basu S Baron-Cohen S Genes related to sex steroids, neural growth, and social-emotional behavior are associated with autistic traits, empathy, and Asperger syndrome Autism Res 2009 2 157 177 10.1002/aur.80 19598235

[B56] Ebstein RP Israel S Chew SH Zhong S Knafo A Genetics of human social behavior Neuron 2010 65 831 844 10.1016/j.neuron.2010.02.020 20346758

[B57] Baron-Cohen S Wheelwright S The Empathy Quotient: an investigation of adults with Asperger syndrome or high functioning autism, and normal sex differences J Autism Dev Disord 2004 34 163 175 15162935 10.1023/b:jadd.0000022607.19833.00

